# Carbapenem-resistant Acinetobacter baumannii (CRAB): metabolic adaptation and transcriptional response to human urine (HU)

**DOI:** 10.21203/rs.3.rs-4415275/v1

**Published:** 2024-05-30

**Authors:** Jenny Escalante, Mase Hamza, Brent Nishimura, Meghan Melecio, Carol Davies-Sala, Marisel R. Tuttobene, Tomás Subils, German M. Traglia, Chloe Pham, Rodrigo Sieira, Luis Actis, Robert A. Bonomo, Marcelo E. Tolmasky, María Soledad Ramirez

**Affiliations:** California State University Fullerton; California State University Fullerton; California State University Fullerton; California State University Fullerton; California State University Fullerton; Universidad Nacional de Rosario; Universidad Nacional de Rosario; Universidad de la República; California State University Fullerton; Fundación Instituto Leloir-IIBBA CONICET; Miami University; Case Western Reserve UniversitySchool of Medicine; California State University Fullerton; California State University Fullerton

**Keywords:** Acinetobacter baumannii, human urine, cefiderocol, human serum albumin, carbapenem-resistance

## Abstract

Carbapenem-resistant *Acinetobacter baumannii* (CRAB) is a major human pathogen and a research priority for developing new antimicrobial agents. CRAB is a causative agent of a variety of infections in different body sites. One of the manifestations is catheter-associated urinary tract infection, which exposes the bacteria to the host’s urine, creating a particular environment. Exposure of two CRAB clinical isolates, AB5075 and AMA40, to human urine (HU) resulted in the differential expression levels of 264 and 455 genes, respectively, of which 112 were common to both strains. Genes within this group play roles in metabolic pathways such as phenylacetic acid (PAA) catabolism, the Hut system, the tricarboxylic acid (TCA) cycle, and other processes like quorum sensing and biofilm formation. These results indicate that the presence of HU induces numerous adaptive changes in gene expression of the infecting bacteria. These modifications presumably help bacteria establish and thrive in the hostile conditions in the urinary tract. These analyses advance our understanding of CRAB’s metabolic adaptations to human fluids, as well as expanding knowledge on bacterial responses to distinct human fluids containing different concentrations of human serum albumin (HSA).

## Introduction

*Acinetobacter baumanniiis* a Gram negative non-fermentative coccobacillus which has emerged as an important human pathogen mainly due to its capacity to persist in hospital settings as well as to resist multiple antimicrobials. Infections with *A. baumannii* are challenging to treat as per an increase in the incidence of multi-drug (MDR) and extensively-drug (XDR) resistant strains^1^. The World Health Organization has placed carbapenem-resistant strains of *A. baumannii* as critical priority for the research and development of new antimicrobials^2^.

*A. baumannii* can cause pneumonia, as well as infections in the bloodstream, skin, soft-tissue, and urinary tract. *Acinetobacter* spp. infections in the clinical setting can be associated to the use of medical devices such as ventilation tubes and central venous and urinary catheters, as well as to surgery, invasive procedures and prolonged treatment with broad spectrum antimicrobials^3^. Studies carried out in intensive care units indicated *A. baumannii* as the main cause of catheter-associated urinary infection (CAUTI) in that setting^4,5^. Up to 20 per cent of all *A. baumannii* isolates are obtained from urinary sources^6^ and recently it was proposed that secondary urinary tract infection (UTI) after re-catheterization could be caused by an intracellular reservoir of *A. baumannii* in bladder epithelial cells in a murine model^7^.

*A. baumannii* has a versatile metabolism that allows it to acquire nutrients, survive and ultimately replicate in a low nutrient environment like the one present in the host during an infection^1,3^. Also, the interaction with the host’s environment can trigger *A. baumannii* metabolic responses that activate antimicrobial resistance and immunomodulatory effects^1,8^. An example of the latter is the catabolism of the alpha amino acid histidine, that in *A. baumannii* is done through the Hut system. The Hut system has an important role in *A. baumannii* infections, as it is implicated in multiple metabolic pathways including zinc homeostasis, biofilm formation, and histamine synthesis^1,9^. Then, studying the physiological responses of *A. baumannii* caused by exposure to environmental conditions that mimic those of the host, is key to understanding its metabolism during infection and, ultimately, for the development of new methods to control those infections^1^.

The catabolism of organic acids has been regarded as essential for the virulence and immunomodulation of *A. baumannii*^1^. Thus, phenylacetic acid (PAA) metabolism plays a key role in *A. baumannii* infection. The PAA catabolic pathway, that is encoded in the *paa* operon, is an important route in the catabolism of aromatic compounds that will lately converge in the Krebs cycle^10,11^. *A. baumannii* mutations in the *ppaE* gene resulted in lesser virulence in a murine septicemia model^10^. Conversely, GacS is a global virulence regulator of *A. baumannii,* and deletion of its gene leads to high repression of the *paa* operon and accumulation of phenylacetate (PA)^8^. In a zebra fish infection model, inhibition of the *A. baumannii paa* operon led to migration of polymorphonuclear neutrophiles to the infection site, with the ultimate consequence of a reduction in bacterial burden and attenuated disease^8^. Another set of experiments demonstrated that subinhibitory concentrations of antimicrobials upregulated the *paa* operon; as well as interfering with PAA metabolism increased susceptibility to antibiotics and hydroxide peroxide treatment^12^. Also, the blockage of PAA catabolism resulted in attenuated virulence in a murine catheter-associated urinary tract infection (CAUTI) model^12^.

Previous results indicated that exposure to human pleural fluid (HPF), a fluid with high content of human serum albumin (HSA), can alter the expression of *A. baumannii* genes related with survival and persistence^13^ and elicit metabolic changes that enhance cytotoxicity and immune evasion^14^ and DNA uptake^15^. Moreover, HPF triggers the differential expression of genes related to processes such as antimicrobial resistance, biofilm formation, motility, osmotic stress, and DNA-damage control, thus acting as an adaptative response to environmental stressors^16^. Still, when *A. baumannii* is exposed to fluids with low HSA content like cerebrospinal fluid (CSF), global changes in gene expression are triggered including the increase of metabolism and virulence expression factors^17^. In this study, we evaluate the transcriptomic response followed by phenotypic analysis to human urine (HU), a fluid low in HSA content, of two carbapenem-resistant *A. baumannii* (CRAB) strains, AB5075^18^ and the AMA40^19^, belonging to different genetic lineages and harboring different cabapenemases^18,19^.

## Results

### Complete metabolic pathways are modulated by human urine (HU)

**Figure 1:**
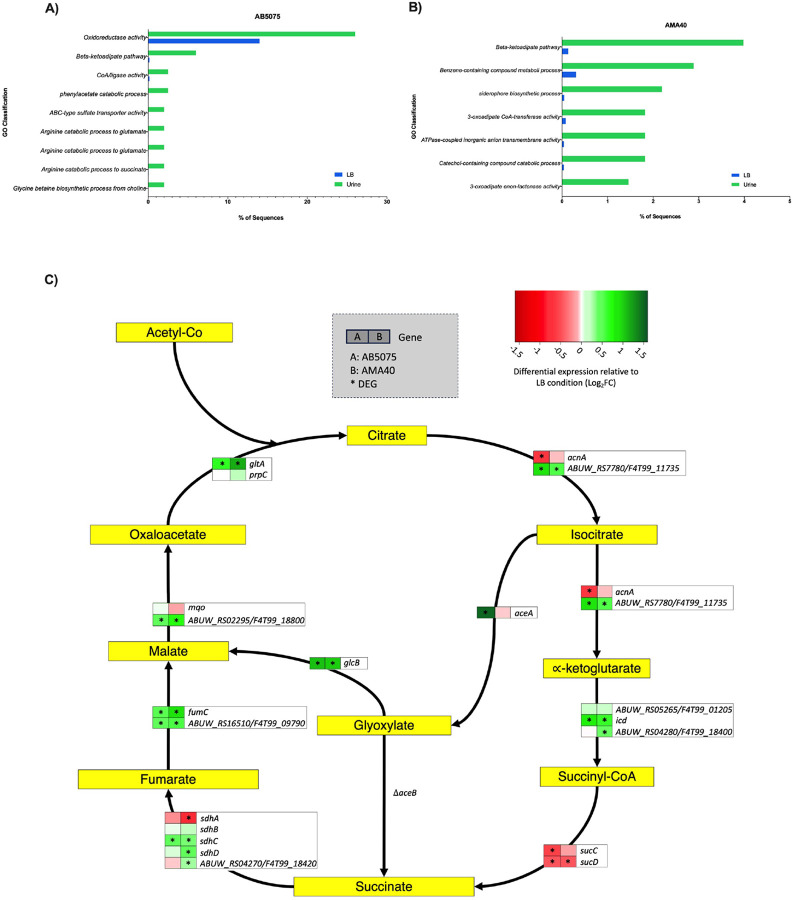
Enriched Gene Ontology terms of the most representative differentially expressed genes. Gene ontology enrichment analysis of the DEGs of strains AB5075 (A) or AMA40 (B) incubated in the presence or in the absence of human urine. Data were obtaining using the Fisher’s test (FDR < 0.05) with the Blast2GO software. C) *A. baumannii* TCA-glyoxylate pathways in response to HU. Reactions and intermediates of the TCA and glyoxylate cycle are represented and based on BioCyc and MetaCyc. Green and red represents up and down-regulation, respectively. *, Indicates statically significant differential expressed genes (DEGs).

RNA-seq analyses were performed using two carbapenem-resistant *A. baumannii* (CRAB) strains, AB5075 and AMA40, belonging to different genetic lineages and possessing different carbapenemases^18^–^19^, in the presence or absence of human urine (HU). RNA-Seq analysis revealed 264 and 455 differentially expressed genes (DEGs) in AB5075 and AMA40, respectively, upon exposure to HU. In presence of HU, AB5075 had 148 genes upregulated, and 116 genes downregulated. In the case of AMA40 strain, 262 genes were upregulated and 193 were downregulated (Supplementary Fig. S1). In terms of gene ontology, the genes corresponded with a variety of functions, such as catalytic activity and metabolic process (Fig. 1A, B). In addition, it was found that of these DEG, 112 were shared by both strains upon HU exposure. Notably, the majority of up- and downregulated genes fell into metabolic pathways. When comparing the data obtained with HU, we observed different results in the expression of genes compared to those obtained with fluids high in HSA or pure HSA^13,14,16,20^.

**Figure 2:**
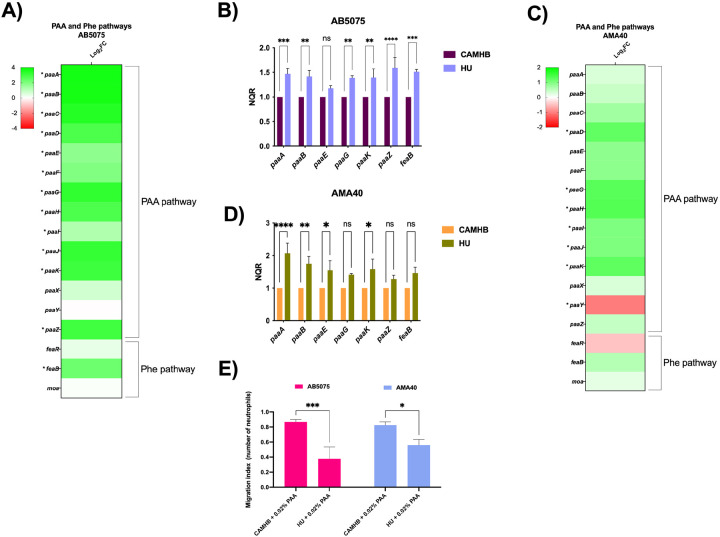
PAA degradation pathway is induced in CRAB by exposure to HU and affects neutrophile chemotaxis. Differential gene expression of genes in PAA and Phe pathways of *A. baumannii* AB5075 when exposed to HU in transcriptomic analyses (A) and by qRT-PCR (B). Differential gene expression of genes in PAA and Phe pathways of *A. baumannii* AMA40 when exposed to HU in transcriptomic analyses (C) and by qRT-PCR (D). Neutrophile chemotaxis assays (E) indicate a reduced neutrophile recruitment when both CRAB strains are exposed to HU. Statistical significance (*P* < 0.05) was determined by two-way ANOVA followed by Tukey’s multiple comparison test. Significance was indicated as follows: ***, *P* <0.001; ** *P* <0.01, and * *P* <0.05.

**Figure 3:**
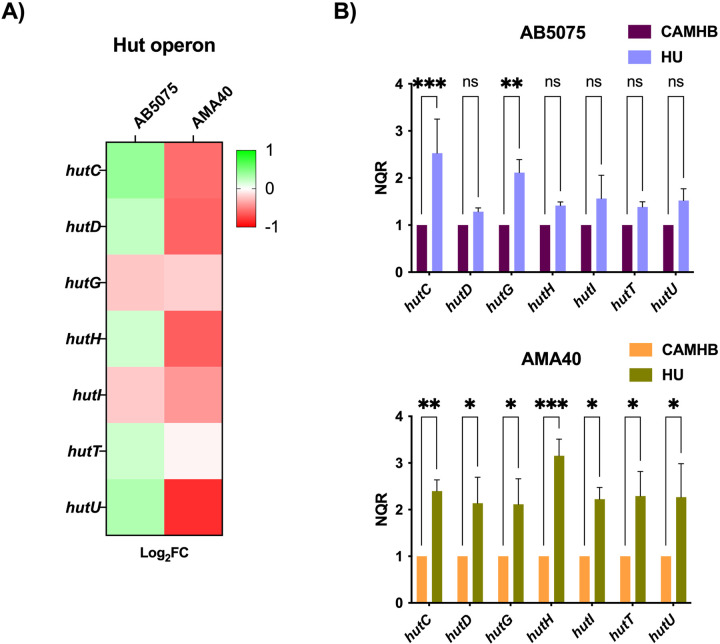
Differential expression of the genes involved on histidine catabolism in CRAB strains exposed to HU. (A) Heatmap outlining the differential expression of genes associated with histidine catabolism for AB5075 and AMA40 strains. (B) qRT-PCR results of the Hut system genes in AB5075 (A) and AMA40 (B). Statistical significance (*P* < 0.05) was determined by two-way ANOVA followed by Tukey’s multiple comparison test. Significance was indicated as follows: *** indicates *p*< 0.001; ** p<0.01, and * p<0.05.

**Figure 4:**
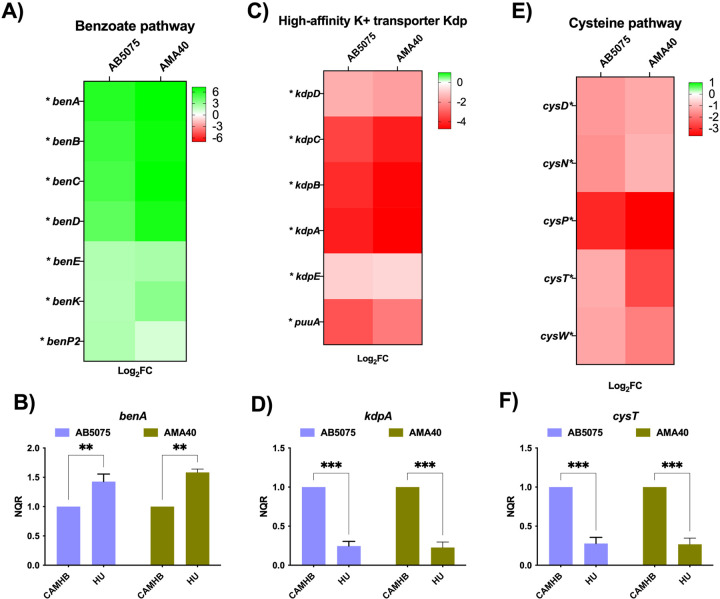
Impact of HU on the expression of CRAB genes coding for K+ transport and benzoate and cysteine pathway functions. Benzoate pathway genes are upregulated in both strains, AB5075 and AMA 40, in transcriptomics studies (A) and corroborated by qRT-PCR (B). Expression of genes involved in the high-affinity potassium transporter are downregulated in both strains of *A. baumannii* studied (C and D). Cysteine metabolic pathway genes are also downregulated in the presence of HU for both strains AB5075 and AMA40 shown by transcriptomic analyses (E) and by qPCR (F). Statistical significance (*P* < 0.05) was determined by two-way ANOVA followed by Tukey’s multiple comparison test. Significance was indicated as follows: *** indicates *P* <0.001; ** *P* <0.01, and * *P* <0.05.

A key pathway is the tricarboxylic acid (TCA) cycle, which is strongly linked to pathogen virulence and has significant influence on energy production, biosynthesis, and adaptation to the host environment. TCA cycle intermediates function as signalling molecules, orchestrating the regulation of virulence genes, and exerting a pivotal impact on host-pathogen interactions, thereby unveiling potential targets for therapeutic interventions^21,22,23, 24^. In both AB5075 and AMA40, a total of 7 out of 18 TCA genes were found to be upregulated. Additionally, 3 out of 18 and 4 out of 18 downregulated TCA genes were identified in AB5075 and AMA40, respectively. Furthermore, in both strains, the glyoxylate pathway exhibited upregulation, with 2 out of 2 and 1 out of 2 genes being upregulated in AB5075 and AMA40, respectively. Notably, the expression of the *aceB* gene was not detected in both strains (Fig. 1C). In addition, genes upregulated in the presence of HU for both *A. baumannii* strains AB5075 and AMA40 correspond to other metabolic pathways such as those for benzoate, acetoin, iron uptake, PAA and Phe catabolic pathways, and the catabolism of central aromatic intermediates (Figs. 2–4 and Supplementary Figs. S1-S4). Regarding genes that are downregulated in association with metabolic pathways in the presence of HU for both strains, these include a decrease in DGE in the high-affinity Potassium transporter Kdp operon (Fig. 4C), as well as in the cysteine (Fig. 4E) and arginine succinyl transferase pathways (Supplementary Fig. S2), along with the taurine and alkanesulfonate transport system pathways (Supplementary Fig. S2). A distinctive difference is observed in the arsenic metabolism where in AMA40 gene expression is clearly downregulated while in AB5075 most genes remain upregulated or slightly downregulated (Supplementary Fig. S2).

Finally, the lipid metabolism was upregulated in both strains under HU condition (Supplementary Fig. S3). These results may indicate that under this condition, which resembles a minimal medium, lipid catabolism is increased. In the following section we describe some of the most relevant pathways affected by the presences of HU in both CRAB studied in this work.

### The PAA degradation pathway of CRAB strains is induced in HU

Considering the relevance of the PAA catabolic pathway in virulence and immune evasion, we decided to assess the expression level of genes involved in the PAA and Phe catabolic pathways in the carbapenem-resistant *A. baumannii* strains AB5075 and AMA40. For these, both CRAB strains were cultured in CAMHB with or without supplementation with HU, and the transcriptome analysis showed that the genes encoding enzymes of the PAA and Phe catabolic pathway are induced under HU in both strains AB5075 (Fig. 2A) and AMA40 (Fig. 2C). Further qRT-PCR experiments confirmed that HU significantly induced the expression of genes of those pathways, such as *paaA, paaB, paaE, paaG, paaK, paaZ* and *feaB* in the strain AB5075 (Fig. 2B). Similar qRT-PCR results were observed in the strain AMA40 (Fig. 2D), confirming that PAA degradation pathway was upregulated by the presence of HU in both CRAB strain evaluated.

It has been shown that if the PAA metabolic pathway is inhibited, the accumulation of metabolic by-products acts as a direct attractant of neutrophils, one of the main immune cells involved in the response to bacterial infections^8^. Therefore, neutrophil chemotaxis assays were performed to evaluate whether HU affects this phenomenon. To this end, both CRAB strains were grown in the presence of PAA, and in the presence or absence of HU. The results obtained were consistent with what was observed at the transcriptional level. Under HU treatment, both strains increased PAA catabolism and attracted significantly fewer neutrophils than the control condition, 2.32-fold and 1.46-fold decrease for AB5075 and AMA40 strains, respectively (Fig. 2E).

### Histidine catabolism gene expression in AB5075 and AMA40 strains is increased under HU condition

The catabolism of the alpha amino acid histidine is done through the Hut system in *A. baumannii*. As mentioned in the introduction, the Hut system is implicated in multiple metabolic pathways that include biofilm formation, zinc homeostasis, and histamine synthesis. The RNA-seq results showed an upregulation in the *hut* operon for AB5075 strain, while for AMA40 strain, the *hutCDGHITU* genes were down-regulated (although not significantly) (Fig. 3A). Also, for both strains, qRT-PCR assays were carried after being cultured in HU respect to CAMHB. Results indicated that the expression of genes linked to histidine catabolism was enhanced in the presence of HU, for AB5075 and AMA40 (Fig. 3B).

### Other metabolic genes of interest in A. baumannii are modulated by HU

As described above in the transcriptomic results, all genes of the benzoate pathway were upregulated when both CRAB strains growth in HU (Fig. 4A). To corroborate this, the expression level of *benA* gene was evaluated by qRT-PCR, resulting in a significant enhanced expression in the presence of HU in both AB5075 and AMA40 (Fig. 4B). Also, RNA-seq results indicated that the expression of high-affinity K^+^ transporter (Kdp) genes was down-regulated in HU conditions (Fig. 4C). In this case, the expression level for *kdpA* gene, was evaluated for RT-qPCR and was obtained as a result a 0.24-fold and 0.22-fold decrease for AB5075 and AMA40, respectively (Fig. 4D). Finally, the cysteine pathway composed of *cysDNPTW* genes was completely repressed under HU conditions in both CRAB strains, according to RNA-seq results (Fig. 4E). To confirm this repression, the evaluation of *cysT* gene was assessed by RT-qPCR, resulting significantly reduced by 5-fold, in both strains, when grown in the presence of HU (Fig. 4F).

### Exposure to human urine affects biofilm formation in CRAB

The lifestyle changes from planktonic to biofilm, and conversely, are key for environmental adaptation and survival of bacteria. Previous studies in animal model indicated that biofilm formation may play a role by increasing virulence in infections by *A*. baumannii^25^. RNA-seq analyses performed in the two carbapenem-resistant *A. baumannii* strains AB5075 and AMA40, showed differential gene expression regarding the genes involved in biofilm formation. Analyses of differentially expressed genes showed that for both strains AB5075 and AMA40 in the presence of HU, genes from the *csu locus* were downregulated. However, in the presence of HU, the genes from the *pga* locus were mostly upregulated in AMA40 while a different pattern can be seen for AB5075 where some genes are slightly downregulated, other slightly downregulated and some show no significant changes (Fig. S4). Biofilm experiments were performed and quantified using crystal violet. As results, a small significant difference was found for the AMA40 strain, while differences were not found in AB5075 between CAMHB and CAMHB + 50% HU conditions (Fig. S4). In addition, CFU recovered from biofilm did not show significant differences (Fig. S4). Finally, the transcriptomic analyses revealed that Quorum Quenching (QQ) related genes were upregulated in the presence of HU in both CRAB strains. While most of Quorum Sensing (QS) associated genes were significantly upregulated or slightly downregulated in AB5075 in presence of HU, they were mostly downregulated in AMA40 (Fig. S4).

## Discussion

CRAB strains can cause CAUTI that are difficult to treat. The ability of *A. baumannii* to survive in a low-nutrient environment, such as the one in the host during an infection, could be explained by the bacterium versatile metabolism. In this work, we have described the effects of exposure to human urine (HU) of two genetically different CRAB strains, AB5075 and AMA40. As a result of HU exposure, we established changes to the differential expression of 264 and 455 genes in strains AB5075 and AMA40, respectively. Further, both strains shared 112 DEGs, suggesting common transcriptional responses to HU.

This common transcriptional response of the studied CRABs to HU exposure includes DEGs in various metabolic pathways. Among these, genes coding for TCA cycle and glyoxylate pathway showed upregulation in both strains, implying potential adaptations in energy production and biosynthesis. In addition, the TCA cycle is linked to the virulence of pathogens through various mechanisms including metabolic adaptation to diverse nutrients allowing biosynthesis and growth, regulation of virulence genes, redox balance, iron acquisition, tissue colonization and immune evasion, and host immune response modulation^21,22^. Another response to HU in both strains tested, is the downregulation of the transcription of genes present in the *kdp* operon, which encodes the high-affinity Potassium transporter Kdp. In E. *coli,* the Kdp transporter is encoded by the *kdpABC* operon, and its expression is regulated by the products of *kdpD* and *kdpE*. Previous observations indicate that the expression of Kdp is affected by the concentration of K^+^ in the medium, resulting in a repression of the operon when the K^+^ concentration in the medium is high^26^. A further study confirmed that in *A. baumannii* the transcription of the five components of the Kdp system is linked, and that *kdpE* is notably upregulated under K^+^ limiting conditions, as well as an important factor for pathogenicity in a murine pneumonia model^27^.

Also, the PAA degradation pathway was induced under HU conditions in both strains, as evidenced by upregulation of genes in the PAA and Phe catabolic pathways. Accordingly, in neutrophil assays, the exposure to HU attracted significantly fewer neutrophils in both CRAB analyzed. These results are consistent with previous observations indicating that PAA metabolism in *A. baumannii* affects infection outcomes by directly influencing neutrophil chemotaxis^8^. Hence, we can conclude that HU is an environmental signal that affects *A. baumannii* metabolism, providing favorable conditions for this pathogen to evade the host immune system. Finally, lipid metabolism was upregulated in both strains under HU conditions, suggesting increased lipid catabolism in a minimal medium-like environment.

It was established that up to 20% of *A. baumannii* clinical isolates are obtained from urinary sources^6^, and CRAB are considered a critical priority for the research and development of new antimicrobials^2^. The findings in this study contribute to a better understanding of metabolic changes undergone by CRAB when exposed to HU. The human fluids that *A. baumannii* may encounter while infecting its host, possess different compositions regarding proteins, metabolites and other solutes. In this sense, a characteristic that distinguishes HU from other fluids is its low content of human serum albumin (HSA).

It was previously reported that *A. baumannii* responds to components of human fluids by modifying its transcriptional and phenotypic profiles^16,17,20^. HSA and human pleural fluid (HPF) can modulate the expression of genes associated with iron uptake systems, biofilm formation, antibiotic resistance, DNA acquisition and metabolism. Previous results indicated that most genes of the *paa* locus were downregulated when exposed to HPF^14^, while as showed in this study *paa* locus is upregulated in presence of HU in both CRAB strains, thus suggesting a differential role of HSA in the regulation of the PAA metabolic pathway. In a similar direction, on *A. baumannii* strain A118 cultured with HSA led to a downregulation of many genes of the *paa* locus with the repressor *paaX* being upregulated^13^.

HSA appears to act as a host-derived signal, inducing adaptive mechanisms that enhance virulence-associated gene expression^49^. Studies on other bacteria like *Bordetella pertussis* and *Pseudomonas aeruginosa* have shown that purified HSA produces outcomes similar to those of serum, and that removing albumin through membrane filtration decreases the serum-mediated effects. This suggests that HSA is the main component responsible for these phenotypes^28,49^.

Additionally, previous research has demonstrated that the effectiveness of cefiderocol, a novel chlorocatechol-substituted siderophore antibiotic used to treat cUTI, is reduced when exposed to human fluids containing HSA, such as Human Serum (HS) and HPF^30,31^. Ferric siderophore transporters facilitate the uptake of cefiderocol into bacterial cells, and the presence of HSA or HSA-containing fluids is associated with a decrease in the expression of genes linked to high-affinity siderophore-mediated iron uptake systems^30^. In contrast, studies have shown that exposure to HU, a fluid with little to no HSA or free-iron content, did not significantly alter the minimum inhibitory concentration (MIC) values for CRABs under the conditions tested^32^. Additionally, genes involved in iron uptake were upregulated. These results support the hypothesis that an unknown mechanism triggers a regulatory response in *A. baumannii* when exposed to human fluids, enabling it to thrive in different environments. Thus, the HSA content in the fluid may play a crucial role in eliciting a differential adaptive response.

In conclusion, this report shows that exposure to HU induces widespread changes in the transcriptome of CRAB strains, impacting various metabolic pathways, as well as genes coding for antibiotic resistance, biofilm formation, and quorum sensing functions. These findings contribute to a better understanding of the adaptive responses of CRAB strains to urinary environments, providing insights that may guide future therapeutic interventions and infection control methods.

## Material and Methods

### Bacterial strains.

Two CRAB strains were used in the present study: the multidrug and hypervirulent AB5075 (OXA-23) strain and AMA40 (NDM-1) (REF).

### RNA extraction.

*A. baumannii* AB5075 and AMA40 were cultured in CAMHB and CAMHB supplement with 50% human urine (HU) from healthy individuals obtained from a certified vendor (Innovative Research Inc., MI, USA) and incubated with agitation for 18 h at 37°C. Then, overnight cultures were then diluted 1:10 in fresh Cation Adjusted Mueller-Hinton Broth (CAMHB), supplemented with HU, and incubated at 37°C with agitation during 7 h. RNA extraction was carried out with Direct-zol RNA Kit (Zymo Research, Irvine, CA, USA), in triplicate. The RNA samples obtained were subjected to DNase treatment (Thermo Fisher Scientific, Waltham, MA, USA) following manufacturer’s instruction, afterwards a PCR amplification of the 16S rDNA gene was performed to confirm there was no DNA contamination. Then, from three independent replicates per sample, ribosomal RNA-depletion was done using the Ribo-Zero kit (Illumina) followed by the construction of the cDNA library with the TruSeq Stranded Total RNA Library Prep kit (Illumina). The RNA sequencing was outsourced to Novogene (Novogene Corporation, Sacramento, CA, USA).

### RNA-seq analysis.

The quality control of the Illumina reads, trimming of low-quality bases and removal of Illumina adapters was performed as described previously (36). Reads were aligned to the genome of *A. baumannii*-AB5075 or AMA40, using Burrows-Wheeler Alignment (BWA) software (v0.7.17) BWA and visualized using the Integrative Genomics Viewer (IGV). Read counts per gene were calculated using FeatureCounts (37). Differential expression analysis was performed using DEseq2 and the Differentially Expressed Genes (DEGs) were defined as those displaying an FDR adjusted *P* value of < 0.05 and log2 fold change > 1.

In addition, to represent metabolic pathways in our RNA sequencing data, the Omics Dashboard Tool provided by both BioCyc^33^ and MetaCyc^34^ was employed. Enrichment or depletion of metabolic pathways was assessed utilizing the Fisher’s exact test hypothesis, with significance determined at *P* values below 0.05. Enrichment or depletion scores (represented as −log_10_ *P* values) for each pathway within the dashboard were subsequently acquired through download and analyzed. The RNA-seq data generated in the current study are available in the NCBI repository with the GEO accession No GSE201259.

### qRT-PCR assays.

The cDNA was prepared using the iScript Reverse Transcription Supermix for qRT-PCR (BioRad, Hercules, CA, USA) and the quantitative PCR was performed using iQ^™^SYBR Green Supermix (BioRad, Hercules, CA, USA), in both cases following the recommendation of the manufacturer. Different primers to confirm RNA-seq results and also study the expression of genes associated with virulence and antimicrobial resistance were used (Table S1). Experiments were performed in technical and biological triplicates. The results were analyzed using the qBASE method^35^ with *recA* and *rpoB* genes as normalizers^36,37^ Data are presented as NRQ (normalized relative quantities). Differences were determined by two-way ANOVA followed by Tukey’s multiple comparison test (*P* < 0.05) using GraphPad Prism (GraphPad software version 10.0.0, San Diego, CA, USA).

### Neutrophil Chemotaxis Assay.

For this assay, a previously published protocol with modifications was used^14^. AB5075 or AMA40 cultured overnight in CAMHB supplement with 50% human urine with or without 0.02% phenylacetic acid (PAA) were tested (Millipore) (2). Controls for this assay constituted of CAMHB and CAMHB + 0.02% PA with performance in parallel. First, 100 μl an overnight culture of *A. baumannii* AB5075 or AMA40 in the tested conditions were combined with 100 μl of chemotaxis buffer (CF). Then, each mix was transferred to a twenty-four-well Olympus polycarbonate tissue culture plate with 8-μm pore size membranes (Genesee Scientific). Prior incubation 100 μl of neutrophils (10 × 10^6^ cells/ml) from iQ Biosciences was added to the semipermeable well inserts. Then, plates were incubated for 1 hour in 5% CO_2_ at 37°C. Neutrophils were then counted from each sample tested. Migration chemotaxis index was calculated by the number of neutrophils in the test well by number of neutrophils in control. All conditions were performed in triplicates. The chemotaxis buffer (CF) has the following components: 25 ml of Roswell Park Memorial Institute Medium (Thermo Fisher), 10% fetal calf serum (FCS) (Thermo Fisher), 500 μl of 100 U/ml penicillin-streptomycin (Sigma Aldrich), and 22 ml of Hanks’ balanced salt solution (HBSS) (Thermo Fisher).

### Biofilm assay.

First, *A. baumannii* AB5075 and AMA40 strains were cultured in fresh CAMHB medium, or CAMHB supplemented with 50% HU in static conditions at 37 °C for 18 h. Then, tubes were emptied, washed three times with 1X phosphate-buffered saline (PBS) and stained with 1% crystal violet (CV) for 15 min. Excess CV was removed by washing three more times with 1X PBS. The biofilm assays were performed in triplicate (absorbance determination at 580 and 660 nm as well CFU/ml), with at least three technical replicates per biological replicate (REF).

### Statistical analysis.

Experiments performed at least in triplicates were statistically analyzed by one- or two-way ANOVA followed by Tukey’s multiple comparison tests using GraphPad Prism (GraphPad software, San Diego, CA, USA). A *P value* < 0.05 was considered significant.

All procedures performed in this study were in accordance with the CSUF Institutional Biosafety Committee Approval plan (DBH117–01) and follow the NIH, CDC, OSHA and other environmental and occupational regulations.

## Data Availability

The datasets generated and analyzed during the current study are available in the Gene Expression Omnibus (GEO) repository (GEO accession No GSE201259).
